# Bladder transitional cell carcinoma anatomic primary site as a predictor of survival and mortality: a population-based retrospective cohort study

**DOI:** 10.1097/MS9.0000000000002581

**Published:** 2024-09-18

**Authors:** Ali Hemade, Souheil Hallit

**Affiliations:** a Faculty of Medicine, Lebanese University, Hadat, Lebanon; b School of Medicine and Medical Sciences, Holy Spirit University of Kaslik, Jounieh, Lebanon; c Applied Science Research Center, Applied Science Private University, Amman, Jordan

**Keywords:** bladder, cancer, epidemiology, survival, mortality

## Abstract

**Background:**

Bladder cancer is a heterogeneous disease with varying prognostic outcomes based on the primary tumor site within the bladder. This study aims to evaluate the impact of tumor location on overall survival and cancer-specific survival in bladder cancer patients.

**Methods:**

The authors conducted a retrospective cohort study using data from the Surveillance, Epidemiology, and End Results database. Patients with primary transitional cell carcinoma of the bladder were categorized based on their tumor locations. Survival outcomes were assessed using Kaplan–Meier analysis and Cox proportional hazards regression models, adjusted for age, sex, race, cancer stage, and treatment modalities. Additionally, binary logistic regression models were employed to predict overall mortality (OM) and cancer-specific mortality (CSM) at 1, 5, and 10 years.

**Results:**

The study included 107 909 patients diagnosed with primary bladder cancer between 2000 and 2021. Significant differences in survival outcomes were observed across different tumor sites. Bladder cancer originating in the urachus had the worst OS before 100 months and the worst CSS overall. Tumors in the anterior wall showed the worst OS after 100 months. In the Cox multivariable analysis, anterior wall tumors were associated with a 1.513-fold increased risk of death compared to lateral wall tumors. The binary logistic regression models showed that anterior wall tumors predicted the highest OM and CSM at 1-year, while urachal tumors had the worst outcomes at 5 and 10 years.

**Conclusions:**

The primary site of bladder cancer is a significant predictor of survival outcomes, with tumors in the urachus and anterior wall associated with a poorer prognosis. These findings underscore the importance of considering tumor location in the prognosis and management of bladder cancer. Future studies should aim to validate these findings in more diverse populations and explore the underlying biological mechanisms that drive these differences.

## Introduction

Highlights

- This study identifies primary bladder tumor location as a critical determinant of overall survival (OS) and cancer-specific survival (CSS).
- Tumors in the anterior wall and urachus exhibit significantly poorer survival outcomes compared to other bladder cancer sites.
- Our findings suggest the need for incorporating tumor location into prognostic models and treatment planning for bladder cancer patients.

Bladder cancer is a significant global health issue, with an estimated 573 000 new cases and 213 000 deaths reported worldwide in 2020 alone^1^. It is the 10th most common cancer globally, with the highest incidence rates observed in Southern and Western Europe, North America, and certain parts of Africa and the Middle East^2^. In the United States, ~81 400 new cases and 17 980 deaths were projected for 2020^3^.

Bladder cancer is a complex and heterogeneous disease that arises from the urothelial cells lining the bladder. It is classified into several subtypes based on histological appearance, with urothelial carcinoma (also known as transitional cell carcinoma) being the most common, accounting for over 90% of cases^4^. The disease is characterized by a high propensity for recurrence, with ~50–70% of nonmuscle invasive bladder cancer patients experiencing recurrence within 5 years of initial treatment^5^. Furthermore, about 10–20% of these patients progress to muscle-invasive bladder cancer, which has a poorer prognosis and requires more aggressive treatment^6^. Bladder cancer is also notable for its association with various risk factors, including tobacco smoking, which is the most significant, responsible for up to 50% of cases^7^. Occupational exposures to certain chemicals, particularly in industries such as dye, rubber, and textile manufacturing, also contribute to the risk, as do chronic urinary tract infections and a history of pelvic radiation^8^. The disease shows a marked male predominance, with men being about four times more likely to be diagnosed than women, though the latter tend to present with more advanced disease at diagnosis and have poorer outcomes^4^.

Bladder cancer prognosis is influenced by a range of factors, including tumor stage, grade, and the presence of specific histological variants. Stage is one of the most critical prognostic factors, with nonmuscle invasive bladder cancer generally having a better prognosis compared to muscle-invasive bladder cancer and metastatic disease^6^. Tumor grade, which reflects the aggressiveness of the cancer cells, also plays a significant role, with high-grade tumors associated with a higher risk of progression and poorer survival outcomes^5^. Additional factors such as lymphovascular invasion, the presence of carcinoma in situ, tumor size, and patient-related factors like age, sex, and comorbidities further refine prognosis^9^. Prognostic scores such as the European Organization for Research and Treatment of Cancer risk tables are widely used to predict the likelihood of recurrence and progression in patients with muscle-invasive bladder cancer and metastatic disease^5^. These scores consider factors such as tumor number, size, prior recurrence rate, and the presence of concomitant carcinoma in situ, providing a valuable tool for individualized patient management. For muscle-invasive bladder cancer, the International Bladder Cancer Nomogram Consortium (IBCN) nomograms are used to predict outcomes postradical cystectomy, incorporating variables such as pathological stage, lymph node involvement, and surgical margins^10^.

The primary site of transitional cell carcinoma within the bladder has not been extensively explored as a potential prognostic factor in the literature. Anatomically, the bladder comprises regions like the trigone, dome, lateral walls, anterior and posterior walls, and bladder neck, with some studies suggesting that tumors in certain areas, such as the bladder neck or trigone, might be associated with poorer outcomes due to their proximity to critical structures like the ureters and urethra, which can complicate surgical management and increase the risk of recurrence or progression^10,11^. For instance, bladder neck tumors have been linked to higher rates of positive surgical margins and lymph node involvement, both of which are adverse prognostic factors^12^. Another study, restricted to outcomes post bladder cancer chemoradiotherapy, described that trigona and bladder neck tumors were associated with increased odds of nodal involvement and worse overall survival after treatment. Beyond that, the notion of the anatomic site of transitional cell carcinoma in the bladder as an independent prognostic factor has not been proven in the literature.

Our study aims to fill this gap by comprehensively analyzing bladder cancer patients categorized by their tumor’s specific anatomic site within the bladder, and the subsequent evaluation of overall mortality (OM) and cancer-specific mortality (CSM) across multiple time intervals (1-year, 5-year, and 10-year). Our goal is to provide more nuanced insights that could influence clinical decision-making and personalized treatment approaches.

## Materials and methods

### Study population

This study is a retrospective cohort analysis of data obtained from the Surveillance, Epidemiology, and End Results (SEER) database, which provides a comprehensive and representative sample covering ~28% of the U.S. population. The cohort consisted of patients who were diagnosed with primary transitional cell carcinoma of the bladder between 2000 and 2021. To ensure the accuracy and relevance of the findings, only patients with a confirmed diagnosis of bladder cancer by histology were included. Individuals diagnosed at autopsy were excluded to avoid introducing bias related to late-stage diagnosis and undetected disease progression, which could confound survival and mortality analysis.

### Data collection

Bladder cancer cases were identified and categorized using the International Classification of Diseases for Oncology, 3rd Edition (ICD-O-3) codes^13^. The specific ICD-O-3 codes utilized in this study were C67.0 (Trigone of bladder), C67.1 (Dome of bladder), C67.2 (Lateral wall of bladder), C67.3 (Anterior wall of bladder), C67.4 (Posterior wall of bladder), C67.5 (Bladder neck), C67.6 (Ureteric orifice), and C67.7 (Urachus). The codes C67.8 (Overlapping lesion of bladder) and C67.9 (Bladder, NOS) were deliberately excluded from the analysis to evaluate and compare the impact of specific primary sites on mortality outcomes.

Demographic and clinical data, including age at diagnosis, sex, race, cancer stage, and treatment modalities such as surgery, chemotherapy, and radiotherapy, were systematically collected. Outcome measures were overall survival (OS), cancer-specific survival (CSS), and overall mortality (OM) and cancer-specific mortality (CSM) at 1-year, 5-year, and 10-year intervals. Our reporting was consistent with the strengthening the reporting of cohort, cross-sectional, and case–control studies in surgery (STROCSS) criteria^14^.

### Statistical analysis

Descriptive statistics were employed to summarize the characteristics of the patient cohort. Continuous variables, such as age and survival time, were reported as means±SD and were compared using Student’s *t*-tests. Categorical variables, including sex, race, and cancer stage, were summarized as frequencies and percentages and analyzed using *χ* ^2^ tests.

To assess survival outcomes, the Kaplan–Meier method was employed to estimate OS and cancer-CSS across different bladder cancer sites^15^. Differences in survival across the primary sites were evaluated using log-rank tests. Furthermore, Cox proportional hazards regression models were applied to identify predictors of survival^16^. This modeling approach, with hazard ratios (HR) and 95% CI calculated for both univariable and multivariable models, provided insights into the relative risk associated with various demographic and clinical factors. Variables with a *P*-value of <0.25 in the univariable analysis were included in the multivariable models^17^.

Binary logistic regression was employed to estimate the likelihood of OM and CSM at different time intervals. The dependent variables in these analyses were OM and CSM, defined for each of the following time intervals: 1-year, 5-year, and 10-year. For overall mortality, any death occurring within the specified time frame, regardless of cause, was coded as 1 (dead). If the patient was alive at the end of the time frame or died after the interval, they were coded as 0 (alive). For cancer-specific mortality, only deaths attributed to bladder cancer were coded as 1 (dead), while all other outcomes (alive or death due to other causes) were coded as 0. The primary site of the tumor was the central predictor variable, with additional covariates including age at diagnosis, sex, race, stage at diagnosis, and treatment modalities.

To evaluate the predictive performance of the models, we calculated the area under the curve (AUC) of the receiver operating characteristic (ROC) curves^18^. Additionally, we calculated the overall accuracy of the models by comparing the predicted outcomes with the observed outcomes.

All statistical analyses were conducted using SPSS version 27, with a *P*-value of <0.05 deemed statistically significant.

### Ethical considerations

This study utilized the SEER database, a publicly available database containing decoded and deidentified data. As such, ethical approval from an institutional review board was not required.

## Results

### Cohort characteristics

Table 1 shows patient characteristics across all selected primary sites. The cohort was 107 909 patients. There was a significant difference in age, with the highest values belonging to the anterior wall site (mean age=71.63 years, *P*<0.0001). We also observed a significant difference in survival time across the primary sites, with the highest values seen in the ureteric orifice site (mean survival time of 84.48 months, *P*<0.0001). There were more males across all primary bladder cancer sites compared to females. White patients has the highest percentage across all primary bladder cancer sites. In regards to staging, only primary bladder cancers originating in the urachus were mostly regional and distant (33.6 and 28.2%, respectivly, *P*<0.0001) while other primary sites were mostly in situ.

**Table 1:** Characteristics of the participants

Primary bladder cancer location	Total	Lateral wall	Anterior wall	Bladder neck	Dome	Posterior wall	Trigone	Urachus	Ureteric orifice	*P*
Characteristics	*n*=107 909	*n*=47 585	*n*=4787	*n*=5859	*n*=7438	*n*=20131	*n*=14209	*n*=411	*n*=7489	
Sex										<0.0001
Males	82 096 (76.1%)	36 356 (76.4%)	3748 (78.3%)	4752 (81.1%)	5723 (76.9%)	15 379 (76.4%)	10 512 (74.0%)	225 (54.7%)	5401 (72.1%)	
Females	25 813 (23.9%)	11 229 (23.6%)	1039 (21.7%)	1107 (18.9%)	1715 (23.1%)	4752 (23.6%)	3697 (26.0%)	186 (45.3%)	2088 (27.9%)	
Race										<0.0001
White	95 866 (88.8%)	42 620 (89.6%)	4159 (86.9%)	5061 (86.4%)	6375 (85.7%)	17 902 (88.9%)	12 673 (89.2%)	306 (74.5%)	6770 (90.4%)	
Black	5379 (5.0%)	2129 (4.5%)	343 (7.2%)	394 (6.7%)	541 (7.3%)	950 (4.7%)	709 (5.0%)	33 (8.0%)	280 (3.7%)	
Other^a^	5706 (5.3%)	2415 (5.1%)	259 (5.4%)	349 (6.0%)	454 (6.1%)	1095 (5.4%)	684 (4.8%)	71 (17.3%)	379 (5.1%)	
Unknown	958 (0.9%)	421 (0.9%)	26 (0.5%)	55 (0.9%)	68 (0.9%)	184 (0.9%)	143 (1.0%)	1 (0.2%)	60 (0.8%)	
Stage										<0.0001
In situ	61 089 (56.6%)	28 543 (60.0%)	1798 (37.6%)	2923 (49.9%)	2833 (38.1%)	11 365 (56.5%)	8150 (57.4%)	24 (5.8%)	5453 (72.8%)	
Localized	35 815 (33.2%)	15 251 (32.1%)	2165 (45.2%)	2093 (35.7%)	3282 (44.1%)	6834 (33.9%)	4447 (31.3%)	123 (29.9%)	1620 (21.6%)	
Regional	6414 (5.9%)	2234 (4.7%)	469 (9.8%)	484 (8.3%)	828 (11.1%)	1085 (5.4%)	935 (6.6%)	138 (33.6%)	241 (3.2%)	
Distant	3513 (3.3%)	1172 (2.5%)	278 (5.8%)	284 (4.8%)	394 (5.3%)	616 (3.1%)	530 (3.7%)	116 (28.2%)	123 (1.6%)	
Unknown	1078 (1.0%)	385 (0.8%)	77 (1.6%)	75 (1.3%)	101 (1.4%)	231 (1.1%)	147 (1.0%)	10 (2.4%)	52 (0.7%)	
Surgery										<0.0001
No	4393 (4.1%)	1556 (3.3%)	248 (5.2%)	274 (4.7%)	363 (4.9%)	1007 (5.0%)	660 (4.7%)	57 (13.9%)	228 (3.0%)	
Yes	10 3350 (95.9%)	45 976 (96.7%)	4518 (94.8%)	5576 (95.3%)	7064 (95.1%)	19 084 (95.0%)	13 522 (95.3%)	354 (86.1%)	7256 (97.0%)	
Chemotherapy										<0.0001
No	80 688 (74.8%)	35 325 (74.2%)	3326 (69.5%)	4453 (76.0%)	5342 (71.8%)	15 224 (75.6%)	10 730 (75.5%)	281 (68.4%)	6007 (80.2%)	
Yes	27 221 (25.2%)	12 260 (25.8%)	1461 (30.5%)	1406 (24.0%)	2096 (28.2%)	4907 (24.4%)	3479 (24.5%)	130 (31.6%)	1482 (19.8%)	
Radiation										<0.0001
No	10 3239 (95.7%)	45 809 (96.3%)	4417 (92.3%)	5513 (94.1%)	6960 (93.6%)	19 336 (96.1%)	13 483 (94.9%)	375 (91.2%)	7346 (98.1%)	
Yes	4670 (4.3%)	1776 (3.7%)	370 (7.7%)	346 (5.9%)	478 (6.4%)	795 (3.9%)	726 (5.1%)	36 (8.8%)	143 (1.9%)	
Sequence number										<0.0001
One primary only	85 968 (79.7%)	38 103 (80.1%)	3853 (80.5%)	4603 (78.6%)	5929 (79.7%)	15 907 (79.0%)	11 392 (80.2%)	359 (87.3%)	5822 (77.7%)	
First of two or more primaries	21 941 (20.3%)	9482 (19.9%)	934 (19.5%)	1256 (21.4%)	1509 (20.3%)	4224 (21.0%)	2817 (19.8%)	52 (12.7%)	1667 (22.3%)	
Age (years±SD)	69.75±12.09	69.44±11.88	71.63±11.95	70.04±12.46	71.49±12.12	70.45±11.98	69.50±12.14	57.29±14.49	67.87±12.37	<0.0001
Survival time (months±SD)	68.12±56.77	69.08±56.70	56.56±52.80	66.08±56.92	60.48±54.57	68.20±56.09	65.22±56.66	58.60±54.48	84.48±59.88	<0.0001

*P*-value <0.05 is statistically significant.

^a^ Other races: American Indian, Asian, Alaska Native, and Pacific Islander.

### Survival analysis

**Figure 1:**
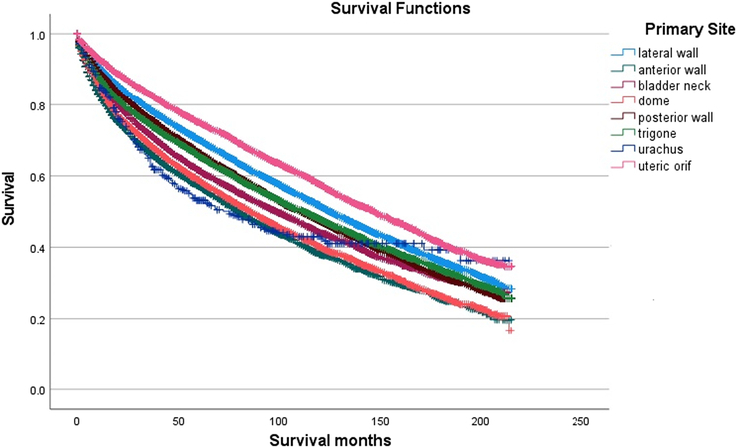
Kaplan–Meier survival curves for overall survival across all primary bladder cancer anatomic sites.

**Figure 2:**
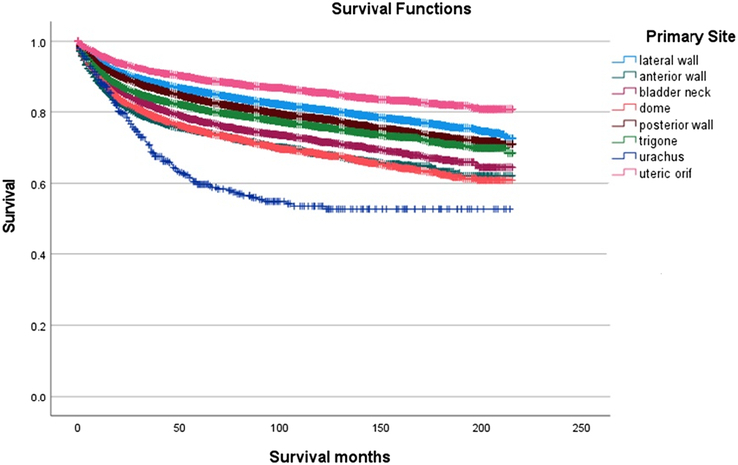
Kaplan–Meier survival curves for cancer-specific survival across all primary bladder cancer anatomic sites.

Patients with bladder cancer in the urachus had the worst OS before 100 months, while bladder cancer of the anterior wall has the worst OS after 100 months (Log-Rank *P*-value <0.0001, Fig. 1). Those with cancer in the urachus had the worst CSS (Log-Rank *P*-value <0.0001, Fig. 2) among the eight sites. As for the other variables, the OS and CSS varied significantly except for OS for sex (Log-Rank *P*-value=0.537).

### Cox univariable and multivariable proportional hazard regression model

The results of the univariable and multivariable OS and CSS models are presented in Table 2. Univariable Cox regression used to predict OS showed that increasing age was a predictor of worse OS (HR=1.069, *P*-value <0.0001, 95% CI=1.068–1.070). Race was also a significant predictor of survival, with white patients having a better OS when taking black race as a reference (0.784, <0.0001, 0.754–0.815 for white race). Stage of bladder cancer was a predictor of survival as well. Tumors described as in situ and localized predicted better survival (0.323, <0.0001, 0.299–0.349 for in situ and 0.625, <0.0001, 0.578–0.675 for localized tumors) while regional and distant tumors predicted significantly worse survival (1.183, <0.0001, 1.090–1.283 for regional and 3.965, <0.0001, 3.645–4.313 for distant tumors). Undergoing surgery predicted better OS (0.592, <0.0001, 0.568–0.616) while receiving chemotherapy and radiotherapy was associated with worse OS (1.099, <0.0001, 1.075–1.123 and 3.397, <0.0001, 3.279–3.519, respectively). Our main variable of interest, anatomic site of primary bladder cancer, was shown to be a significant predictor of OS. Specifically, all primary sites exibited worse OS when compared to bladder tumors of the lateral wall except for bladder cancer originating in the ureteric orifice (0.841, <0.0001, 0.809–0.873), with the worse OS predicted by primary cancer originating in the anterior wall (1.513, <0.0001, 1.451–1.578). Multivariable Cox regression to predict OS showed comparable trends to the univariable model across variables mentioned above, except the fact that receiving chemotherapy was associated with better OS (0.869, <0.0001, 0.849–0.890). Primary bladder cancer anatomic sites were significant predictors of worse OS except for the dome of the bladder, the urachus, and the ureteric orifice.

**Table 2:** Univariable and Multivariable Cox proportional hazards regression models for predictors of overall survival and cancer-specific survival in the analytic cohort

	Overall survival						Cancer-specific survival					
	Univariable Cox model			Multivariable Cox model			Univariable Cox model			Multivariable Cox model		
Characteristics	HR	*P*	95% CI	HR	*P*	95% CI	HR	*P*	95% CI	HR	*P*	95% CI
Age (years)	1.069	**<0.0001**	1.068–1.070	1.068	**<0.0001**	1.067–1.070	1.045	**<0.0001**	1.044–1.047	1.045	**<0.0001**	1.043–1.046
Sex												
Males	1						1			1		
Females	1.007	0.538	0.986–1.028				1.256	**<0.0001**	1.217–1.297	1.105	**<0.0001**	1.070–1.142
Race												
Black	1			1			1			1		
Other^a^	0.676	**<0.0001**	0.638–0.715	0.620	**<0.0001**	0.586–0.657	0.585	**<0.0001**	0.538–0.636	0.618	**<0.0001**	0.568–0.672
Unknown	0.159	**<0.0001**	0.126–0.201	0.213	**<0.0001**	0.168–0.268	0.085	**<0.0001**	0.054–0.132	0.148	**<0.0001**	0.095–0.230
White	0.784	**<0.0001**	0.754–0.815	0.767	**<0.0001**	0.737–0.798	0.608	**<0.0001**	0575–0.643	0.713	**<0.0001**	0.674–0.755
Primary site												
Lateral wall	1			1			1			1		
Anterior wall	1.513	**<0.0001**	1.451–1.578	1.084	**<0.0001**	1.039–1.131	1.873	**<0.0001**	1.760–1.993	1.110	**0.001**	1.042–1.182
Bladder neck	1.255	**<0.0001**	1.207–1.306	1.068	**0.001**	1.027–1.111	1.582	**<0.0001**	1.491–1.677	1.178	**<0.0001**	1.111–1.250
Dome	1.421	**<0.0001**	1.372–1.471	1.024	0.186	0.989–1.061	1.822	**<0.0001**	1.731–1.918	1.094	**<0.0001**	1.039–1.153
Posterior wall	1.130	**<0.0001**	1.102–1.158	1.037	**0.005**	1.011–1.063	1.159	**<0.0001**	1.113–1.208	1.040	0.060	0.998–1.084
Trigone	1.144	**<0.0001**	1.112–1.177	1.081	**<0.0001**	1.051–1.113	1.340	**<0.0001**	1.282–1.401	1.178	**<0.0001**	1.127–1.232
Urachus	1.379	**<0.0001**	1.199–1.586	0.888	0.098	0.771–1.022	2.747	**<0.0001**	2.338–3.227	0.810	**0.012**	0.688–0.954
Ureteric orifice	0.841	**<0.0001**	0.809–0.873	0.990	0.594	0.952–1.028	0.732	**<0.0001**	0.683–0.784	0.959	0.238	0.895–1.028
Stage												
Unknown	1			1			1			1		
In situ	0.323	**<0.0001**	0.299–0.349	0.417	**<0.0001**	0.384–0.452	0.125	**<0.0001**	0.112–0.140	0.180	**<0.0001**	0.160–0.202
Localized	0.625	**<0.0001**	0.578–0.675	0.739	**<0.0001**	0.681–0.802	0.577	**<0.0001**	0.518–0.643	0.759	**<0.0001**	0.676–0.852
Regional	1.183	**<0.0001**	1.090–1.283	1.580	**<0.0001**	1.449–1.723	1.636	**<0.0001**	1.464–1.828	2.353	**<0.0001**	2.088–2.652
Distant	3.965	**<0.0001**	3.645–4.313	5.440	**<0.0001**	4.979–5.943	5.539	**<0.0001**	4.949–6.201	7.552	**<0.0001**	6.699–8.513
Surgery												
No	1			1			1			1		
Yes	0.592	**<0.0001**	0.568–0.616	0.717	**<0.0001**	0.684–0.748	0.438	**<0.0001**	0.414–0.464	0.610	**<0.0001**	0.575–0.648
Chemotherapy												
No	1			1			1			1		
Yes	1.099	**<0.0001**	1.075–1.123	0.869	**<0.0001**	0.849–0.890	1.549	**<0.0001**	1.501–1.598	0.877	**<0.0001**	0.848–0.908
Radiation												
No	1			1			1			1		
Yes	3.397	**<0.0001**	3.279–3.519	1.480	**<0.0001**	1.424–1.537	5.199	**<0.0001**	4.978–5.429	1.533	**<0.0001**	1.481–1.627
Sequence number												
One primary only	1			1			1			1		
First of two or more primaries	1.074	**<0.0001**	1.052–1.097	1.102	**<0.0001**	1.079–1.125	0.854	**<0.0001**	0.824–0.885	0.929	**<0.0001**	0.896–0.964

Numbers in bold indicate significant *P*-values.

^a^ Other races: American Indian, Asian, Alaska Native, and Pacific Islander.

HR, Hazard ratio.

Predicting CSS, the univariable Cox regression model showed similar predictors compared to OS, such as age at diagnosis, stage, receiving chemotherapy, undergoing surgery at the cancer site, and receiving radiotherapy. Sex, which did not predict OS, was a significant predictor of CSS, and females showed worse CSS compared to males (1.256, <0.0001, 1.217–1.297). Primary bladder cancer anatomic site continued to be a significant predictor of survival from death specifically caused by the primary bladder cancer diagnosis. However, cancer originating in the urachus predicted worse CSS (2.747, <0.0001, 2.338–3.227) compared to the OS model, which predicted OS to be the worst in cancers originating in the anterior wall of the bladder. As for the multivariable CSS model, similar trends were found compared to the OS multivariable model with the addition of sex being a significant predictor of CSS but not OS. Primary bladder cancer anatomic site continued to be a significant predictor of CSS with the exception of posterior wall and ureteric orifice. The worst survival in the multivariable CSS model was predicted by diagnosis of a tumor in the bladder neck (1.178, <0.0001, 1.111–1.250).

### Binary logistic regression models

Table 3 represents the results of the multiple binary logistic regression models. Our main variable of interest was primary bladder cancer anatomic site, also including confounders (sex, stage, race, receiving chemotherapy, receiving radiotherapy, undergoing surgery, and sequence number). Table 4 shows the accuracy measures of the models. The anterior wall of the bladder site predicted the highest OM and CSM in the 1-year interval. Tumors of the Urachus had the worst OM in the 5-year and 10-year models and the worst 10-year CSM. The worst 5-year CSM was predicted by being diagnosed with cancer in the bladder neck. All of our models exhibited AUC values above 0.75. The model with consistently the highest AUC-accuracy measures was the 5-year CSM model.

**Table 3:** Odds ratios and *P*-values for 1-year, 5-year, and 10-year OM and CSM for the different primary bladder cancer anatomic site for the cohort using BLR

	1-year OM		5-year OM		10-year OM		1-year CSM		5-year CSM		10-year CSM	
	OR	*P* [95% CI]	OR	*P* [95% CI]	OR	*P* [95% CI]	OR	*P* [95% CI]	OR	*P* [95% CI]	OR	*P* [95% CI]
Lateral wall	1		1		1		1		1		1	
Anterior wall	1.302	**<0.0001** [1.188–1.426]	1.187	**<0.0001** [1.105–1.275]	1.192	**<0.0001** [1.110–1.281]	1.381	**<0.0001** [1.238–1.540]	1.256	**<0.0001** [1.151–1.369]	1.223	**<0.0001** [1.126–1.328]
Bladder neck	1.131	**0.008** [1.033–1.238]	1.185	**<0.0001** [1.108–1.268]	1.152	**<0.0001** [1.078–1.232]	1.330	**<0.0001** [1.194–1.481]	1.319	**<0.0001** [1.214–1.433]	1.291	**<0.0001** [1.194– 1.396]
Dome	1.088	**0.035** [1.006–1.177]	1.102	**0.001** [1.039–1.169]	1.095	**0.003** [1.032–1.161]	1.191	**<0.0001** [1.084–1.309]	1.264	**<0.0001** [1.176–1.358]	1.251	**<0.0001** [1.169–1.339]
Posterior wall	1.009	0.758 [.951–1.072]	1.067	**0.002** [1.023–1.112]	1.073	**0.001** [1.030–1.117]	1.032	.409 [0.957–1.114]	1.069	**.018** [1.012–1.130]	1.057	**.034** [1.004–1.113]
Trigone	1.193	**<0.0001** [1.117–1.273]	1.162	**<0.0001** [1.107–1.218]	1.133	**<0.0001** [1.082–1.187]	1.319	**<0.0001** [1.218–1.429]	1.285	**<0.0001** [1.209–1.365]	1.237	**<0.0001** [1.168–1.310]
Urachus	0.618	**0.002** [0.453–0.842]	1.453	**0.002** [1.151–1.834]	1.634	**<0.0001** [1.290–2.070]	0.573	**0.001** [0.409–0.803]	1.227	0.101 [0.961–1.566]	1.324	**0.021** [1.044–1.678]
Ureteric orifice	0.923	0.120 [.834–1.021]	0.983	0.599 [0.920–1.049]	0.986	0.655 [0.928–1.048]	0.853	**0.021** [0.745–0.976]	0.940	0.191 [0.857–1.031]	0.922	0.06 [0.847–1.003]

Numbers in bold indicate significant *P*-values.

BLR, binary logistic regression; CSM, cancer-specific mortality; OM, overall mortality; OR, odds ratio.

**Table 4:** Area under the curve and accuracy values for the 1-year, 5-year, and 10-year overall mortality and cancer-specific mortality models

	1-year OM	5-year OM	10-year OM	1-year CSM	5-year CSM	10-year CSM
AUC	0.752996	0.821704	0.850189	0.786157	0.815890	0.822150
Accuracy	0.894253	0.778424	0.757592	0.928263	0.872994	0.854182

AUC, area under the curve; CSM, cancer-specific mortality; OM, overall mortality.

## Discussion

The present study provides a comprehensive analysis of bladder cancer with a particular focus on the prognostic significance of primary tumor sites within the bladder. Our findings reveal substantial differences in OS and CSS based on tumor location, contributing new insights into an area that has been underexplored in the literature.

One of the key findings of our study is the significant variation in OS and CSS among different bladder cancer primary sites. Specifically, we found that bladder cancers originating in the urachus and anterior wall were associated with the worst survival outcomes. The poor prognosis associated with urachal tumors has been documented in prior studies, often attributed to their deep-seated location and delayed presentation, which typically occurs at a more advanced stage^19,20^, which was the case in our study, as most tumors of the urachus were diagnosed in the regional and distant stage. These two studies were based on very few cases (42 and 46, respectively). Urachal tumors are often difficult to diagnose early, leading to a more aggressive disease course and poorer outcomes^21^. In our study, urachal bladder cancers had a significantly younger age at presentation (57.29 years) compared to the total cohort (69.75 years), challenging previous findings^19,20^. However, the difference in sample size likely led to this finding. As for the anterior wall site, it was less commonly discussed in the literature as a bladder cancer location with particularly poor outcomes, making our finding of the worst OS after 100 months a novel contribution. This observation may suggest that tumors in the anterior wall could possess unique biological characteristics or respond differently to treatment over time. It is also possible that the anterior wall’s anatomical location may pose challenges in complete surgical resection or contribute to a different pattern of disease progression, though further research would be necessary to confirm these hypotheses.

Our results also show that the primary site within the bladder was a significant predictor of both OS and CSS in univariable and multivariable Cox regression models. This finding is consistent with reports that suggest anatomical location within the bladder can influence the prognosis due to factors like tumor accessibility during surgery^11^. In addition to the anterior wall of the bladder and the urachus sites predicting the worse outcomes, we found that bladder neck tumors predicted the worse CSS, which resonates with a previous study that showed that bladder neck tumors are associated with a higher risk of positive surgical margins and lymph node involvement, both of which are adverse prognostic factors^12^. We also observed that receiving chemotherapy was associated with improved OS in the multivariable model, despite an initial association with worse OS in the univariable analysis. This discrepancy may be explained by the fact that patients receiving chemotherapy are often those with more advanced disease or poor prognosis, which could explain the worse outcomes seen in the univariable analysis. Once other confounding factors, such as disease stage, race, and surgery are controlled for in the multivariable model, the beneficial effects of chemotherapy become apparent. This finding is consistent with existing literature, which supports the use of chemotherapy in advanced bladder cancer to improve survival outcomes^22,23^.

The binary logistic regression models in our study, which predicted OM and cancer-specific mortality CSM at 1, 5, and 10 years, further emphasize the prognostic significance of the primary tumor site. The finding that tumors in the anterior wall predict the highest OM and CSM at 1-year, while urachal tumors have the worst outcomes at 5 and 10 years, highlights the temporal dynamics of bladder cancer prognosis. This suggests that certain tumor locations may exert their effects on survival at different stages of the disease course. Interestingly, the survival advantage seen in ureteric orifice tumors compared to other sites, as indicated by the lower hazard ratios for OS, is another novel finding that contrasts with earlier studies that have not specifically highlighted this site. It is possible that tumors in this location are diagnosed earlier due to symptoms like hematuria or are more amenable to complete surgical resection, leading to better outcomes^24,25^. However, further research is required to substantiate this finding.

### Limitations

This study, while offering important insights into the prognostic significance of primary bladder cancer sites, is subject to several limitations that should be considered when interpreting the findings. Firstly, the retrospective design of the study introduces inherent biases related to data collection and reporting. Retrospective studies rely on the accuracy and completeness of existing medical records, which may result in misclassification or underreporting of key variables, such as tumor characteristics, treatment details, and patient outcomes. Although the data were obtained from a comprehensive and reputable database, the potential for inaccuracies or missing information remains. Secondly, our cohort, primarily representing specific demographic and geographic areas of the United States, may not fully reflect the broader population of bladder cancer patients globally. This is particularly relevant given the known racial and ethnic disparities in bladder cancer incidence, presentation, and outcomes. Thirdly, the study did not account for certain potentially influential variables, such as genetic or molecular markers, and clinical, laboratory, and therapeutic regimen details, which are increasingly recognized as important in the prognosis and treatment of bladder cancer.

### Relevance and implications

The relevance and implications of this study are significant for both clinical practice and future research in the management of bladder cancer. By identifying primary tumor location within the bladder as a critical factor influencing OS and CSS, this study challenges the traditional approach that often overlooks the anatomical site as a prognostic variable. The finding that tumors in the anterior wall and urachus are associated with particularly poor outcomes underscores the need for more tailored treatment strategies that take tumor location into account. These insights could lead to the development of more accurate prognostic models, helping clinicians better predict patient outcomes and optimize therapeutic interventions. On the other hand, several future research directions are warranted to build upon these findings and enhance our understanding of bladder cancer prognosis. Future studies should aim to validate our findings in multinational cohorts that include more diverse populations. Prospective studies, which offer more controlled data collection, would be particularly valuable in minimizing biases. Incorporating genetic and molecular profiling into future research could provide deeper insights into the biological mechanisms driving the observed differences in survival based on tumor location. Specifically, it is desirable for future research to look for clinical and pathologic similarities among bladder tumors originating in the anterior wall, urachus, and bladder neck, which were the sites associated with the worst survival in our study.

## Conclusions

This study highlights the significant prognostic impact of the primary tumor site in bladder cancer, demonstrating that the location of the tumor within the bladder plays a crucial role in determining both OS and CSS. Our findings suggest that tumors originating in sites such as the urachus and anterior wall, are associated with poorer survival outcomes compared to more common sites, like the lateral wall. These results emphasize the importance of considering tumor location when assessing patient prognosis and planning treatment strategies. While the study provides novel insights, particularly regarding the prognostic significance of anterior wall tumors, it also raises questions about the underlying biological mechanisms that contribute to the observed differences in survival.

## Ethical approval

As this study involves retrospective analysis of data deidentified and decoded by the Surveillance, Epidemiology, and End Results (SEER) Program and did not involve any direct or indirect patient contact or intervention, an institutional review board (IRB) approval was not required.

## Consent

Our study did not involve any direct or indirect human contact. We utilized a publicly available decoded database (SEER). This is in line with the guidelines by the OHRP. https://www.hhs.gov/ohrp/regulations-and-policy/guidance/research-involving-coded-privateinformation/index.html.

## Source of funding

Not applicable.

## Author contribution

A.H.: conceptualization, data curation, investigation, methodology, and writing – original draft; S.H. and A.H.: formal analysis; S.H.: supervision and writing – review. All writers agreed on the final manuscript.

## Conflicts of interest disclosure

The authors declare no conflicts of interest.

## Research registration unique identifying number (UIN)

This study did not involve any human participants whatsoever. Data was sourced from the SEER database, available online, containing deidentified data with no chance of tracing back patients. Our study is not eligible for registration.

## Guarantor

Ali Hemade.

## Data availability statement

Data utilized in our study is publicly available as part of the SEER database.
